# Optimized R functions for analysis of ecological community data using the R virtual laboratory (RvLab)

**DOI:** 10.3897/BDJ.4.e8357

**Published:** 2016-11-01

**Authors:** Constantinos Varsos, Theodore Patkos, Anastasis Oulas, Christina Pavloudi, Alexandros Gougousis, Umer Zeeshan Ijaz, Irene Filiopoulou, Nikolaos Pattakos, Edward Vanden Berghe, Antonio Fernández-Guerra, Sarah Faulwetter, Eva Chatzinikolaou, Evangelos Pafilis, Chryssoula Bekiari, Martin Doerr, Christos Arvanitidis

**Affiliations:** ‡ Institute of Computer Science, Foundation of Research and Technology Hellas, Heraklion, Greece; § Institute of Marine Biology, Biotechnology and Aquaculture, Hellenic Centre for Marine Research, Heraklion, Crete, Greece; | Department of Biology, University of Ghent, Ghent, Belgium, Department of Microbial Ecophysiology, University of Bremen, Bremen, Germany; ¶ University of Glasgow, Glasgow, United Kingdom; # Vrije Universiteit Brussels, 1050, Brussels, Belgium; ¤ University of Oxford, Oxford e-Research Centre, Oxford, United Kingdom

**Keywords:** Parallel data manipulation, R, *pbdMPI* package, Single Program Multiple Data (SPMD) parallelization, virtual enviroment, *vegan* package, biodiversity analyses, ecological analyses

## Abstract

**Background:**

Parallel data manipulation using R has previously been addressed by members of the R community, however most of these studies produce *ad hoc* solutions that are not readily available to the average R user. Our targeted users, ranging from the expert ecologist/microbiologists to computational biologists, often experience difficulties in finding optimal ways to exploit the full capacity of their computational resources. In addition, improving performance of commonly used R scripts becomes increasingly difficult especially with large datasets. Furthermore, the implementations described here can be of significant interest to expert bioinformaticians or R developers. Therefore, our goals can be summarized as: (i) description of a complete methodology for the analysis of large datasets by combining capabilities of diverse R packages, (ii) presentation of their application through a virtual R laboratory (RvLab) that makes execution of complex functions and visualization of results easy and readily available to the end-user.

**New information:**

In this paper, the novelty stems from implementations of parallel methodologies which rely on the processing of data on different levels of abstraction and the availability of these processes through an integrated portal. Parallel implementation R packages, such as the *pbdMPI* (Programming with Big Data – Interface to MPI) package, are used to implement Single Program Multiple Data (SPMD) parallelization on primitive mathematical operations, allowing for interplay with functions of the *vegan* package. The *dplyr* and *RPostgreSQL* R packages are further integrated offering connections to dataframe like objects (databases) as secondary storage solutions whenever memory demands exceed available RAM resources.

The RvLab is running on a PC cluster, using version 3.1.2 (2014-10-31) on a x86_64-pc-linux-gnu (64-bit) platform, and offers an intuitive virtual environmet interface enabling users to perform analysis of ecological and microbial communities based on optimized *vegan* functions.

A beta version of the RvLab is available after registration at: https://portal.lifewatchgreece.eu/

## Introduction

The advent of interdisciplinary science fields like computational ecology/biodiversity and metagenomics (Oulas et al. 2015, Canhos et al. 2004, Petrovskii and Petrovskaya 2012, Soberon and Peterson 2004) is contributing to the constant escalation of complex computational pipelines, which, in turn, requires increased computational resources and capacities. The size and speed of the computational analyses are limited by the source code which delineates the accessible functions and libraries. The ever growing in popularity and usability R statistical programming language (R Core Team 2013) provides a wide array of built-in functions, libraries and packages that are of valuable use to the environmental ecologist, microbiologist as well as many other academic disciplines. The use of these functions is often sub-optimal with respect to data size manipulation and speed-up. However, the average biologist is often not inclined to become acquainted with the necessary programming and information technology (IT) skills, required to efficiently transform conventional available functions into computationally optimized methods. Therefore, they are deprived from speed-up and improved memory manipulation during their computational and mathematical operations.

On the other hand, computer scientists are well-aware of tools, methods and implementations that can provide significant boosts in speed for computational calculations and further solve issues like memory exhaustion, a problem often faced in analyses using “big” data.

In this work, we have brought together expert scientists from the disciplines of environmental ecology and microbiology with IT and mathematical experts in order to focus on optimization methods for widely used statistical functions, effective in environmental ecology today. More specifically, we focus on the *vegan* (Community Ecology) package (Oksanen et al. 2015) available in R and the ways to optimise common functions with respect to both speed-up and memory usability. This work comes together under a virtual laboratory (vLab) which is available through the LifeWatchGreece portal.

Similar efforts, as in Buttigieg and Ramette (2014), have resulted in the creation of online R platforms, such as the "Multivariate AnalysiS Applications for Microbial Ecology (MASAME)" suite; it seems that there is a need for the creation of such platforms as more and more scientists are leaning towards the use of open source software for their analyses. However, although MASAME makes use of R and some of the *vegan* functions are available for the users, there is no extra effort on their optimization and parallelization.

Our main incentive is to make optimization tasks easily available to the average user who has no expertise and prior training in this area of research. This way, environmental ecologists can make use of optimized functions, implemented by IT experts and mathematicians, through a freely available, user-friendly interface, without having to spend time analysing parallelization complexity and deciding on which function to use and how to do so. In addition, multiple non-parallelized functions are also available for users with no programming experience via the RvLab interface. Source code and methodologies are accessible to users with programming and IT knowledge.

We describe the optimization methods and their implementation in detail and highlight the advantages of using our optimized R functions, with respect to both computational time speed-up, as well as improved memory manipulation in order to avoid memory exhaustion issues. The analyses we focus on can be computationally demanding primarily due to large matrix operations, increasing permutations in likelihood function computations and iterative basic mathematical operations.

### Motivation and State-of-the-Art

Despite its popularity among the research community, R still seems inflexible in fully exploiting the latest developments in computer software and hardware. As there are no inherent constructs for parallelizing computations, it is up to the developer to adapt the R code, in order to take advantage of the resources available by multi-core CPUs. Moreover, when the data computed are too big to fit in main memory, no simple solution is considered standard. Packages that offer workarounds are indeed available, but their use by non-expert R users is rarely considered straightforward. These are real issues faced by researchers, whose needs for processing collected data continually increase both in computational demand and in size.

Working with large datasets in R can be cumbersome because of the need to keep objects in physical memory. The need to keep whole objects in memory becomes a challenging task to those who might want to work interactively with large datasets. Several packages attempt to overcome problems when accessing big volumes of data. The *bigmem* package (Kane et al. 2013) is designed to handle massive data sets that are not larger than the available RAM. It overcomes the restriction of R using matrices or data frames that, even though they fit in RAM, no space is available to handle the overhead of working with them. Furthermore, it extends and augments the R statistical programming environment, thus enabling more powerful parallel analyses and data mining of massive data sets, although it is restricted to the available RAM size (even though some options for connecting its objects with file-backed mappings can be implemented).

The Programming with Big Data in R (pbdR) project (Ostrouchov et al. 2012) seeks to elevate the R language to supercomputers. Most of its functionalities revolve around parallelization features, yet the *pbdDMAT* package (Schmidt et al. 2012) of the project offers an implicitly parallel system for doing distributed matrix computation in R. The *bigmem* and *pbdDMAT* packages are useful solutions, but do not always provide the level of flexibility needed in handling complex constructs because of the fact that they rely on their own constructs to handle big data.

Nowadays, the *dplyr* package (Wickham and Francois 2015) has become very popular for data manipulation, providing a repertoire of functions for accessing data stored in databases. Coupled with packages dedicated for specific database implementations, such as *RPostgreSQL* (Conway et al. 2013), it offers the possibility to write R scripts that access the underlying databases from within the R environment, but with the look and feel of relational data manipulation. This approach is more attractive for a large scale implementation, such as the one designed for the LifeWatchGreece portal.

As far as parallelization is concerned, a multitude of packages have emerged, such as *snow* (Tierney et al. 2015), *multicore* (Urbanek 2009) and *parallel*. Due to the complexity of tasks required in *vegan*, as well as in other packages for the LifeWatchGreece project, we found the existing approaches for parallel computing rather restrictive. Our decision in this project was to go into the low-level whenever necessary and implement custom parallel solutions which can provide more flexibility. To assist us in this process, packages that provide interfaces to MPI (Message-Passing Interface) for R are proved really valuable. *Rmpi* (Yu 2002) is one of the most popular solutions that can port low level MPI functions into R, abstracting the complexities of writing C or Fortran code. The more recent *pbdR* project (Ostrouchov et al. 2012) also offers such a wrapper through the *pbdMPI* library, which is intended for Single Program Multiple Data (SPMD) programming with big data.

After considering the benefits offered, we decided to adopt *pbdMPI* (Chen et al. 2012, as our primary package for parallelization within LifeWatchGreece, and couple it with other solutions for parallelization or optimization of code, whenever necessary.

Summarizing, the work conducted in the context of the project aims at applying optimization techniques for data on two different levels of abstraction described in detail in the project description. This is done by:

1) Using the *pbdMPI* package to implement Single Program Multiple Data (SPMD) parallelization on primitive mathematical operations, allowing for interplay with functions of the *vegan* package.

2) Using the *dplyr* and *RPostgreSQL* packages in order to offer secondary storage solutions whenever memory demands exceed available RAM resources (memory exhaustion)

Option (1) may be employed in conjugation with option (2) to address memory exhaustion issues. While, for speed-up and job segmentation issues we only use option (1).

We finally evaluate our optimization results using two test case scenarios with real data obtained from environmental ecologists in the standard file formats, commonly utilized in the field. We also demonstrate results and visualization outcomes obtained through the graphical user interface available through the LifeWatchGreece portal.

## Project description

### Design description

**Figure 1.**
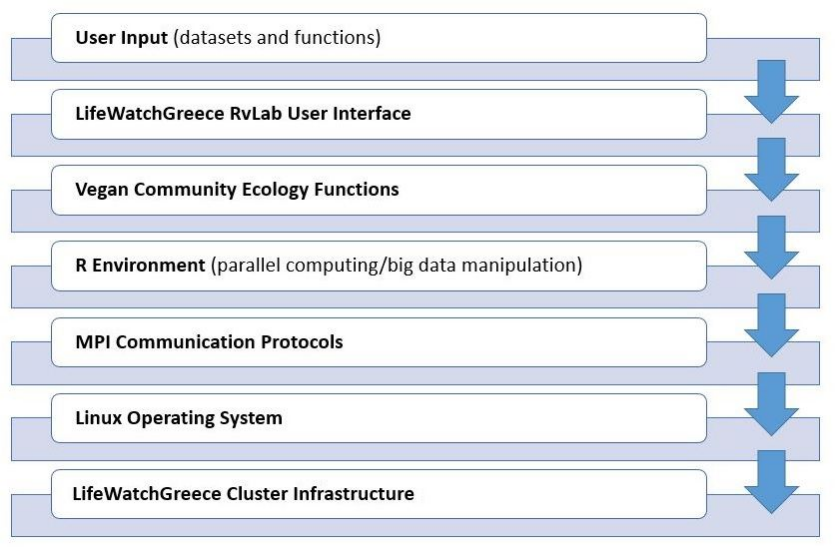
The general architecture design to approach the development of the LifeWatchGreece RvLab.

The general architecture design to approach the development of the LifeWatchGreece RvLab is presented in Fig. 1. At the bottom layer, a dedicated multi-core cluster has been installed, providing the necessary resources for supporting the execution of demanding computational tasks (jobs) submitted by RvLab users. On top of that, all jobs are inserted into a priority queue and forwarded to the cluster within the Linux Operating System.

Each R script communicates with the cluster using the MPI message-passing protocol. RvLab abstracts the implementation details from the end-user: each *vegan* function implemented for execution within the RvLab takes advantage of the appropriate R packages for parallel computing and big data manipulation, which are preconfigured to adapt to the workload of the cluster at each particular moment.

An intuitive User Interface provides all necessary facilities for end users to perform ordinary tasks, such as to upload their datasets, to choose and parameterize the desirable *vegan* functions available by the RvLab, to monitor the progress of execution of their submitted jobs, and to visualize and download the produced results.

**Optimization Analysis Process**

The core functionalities of the RvLab lie in the middle layers of this architecture, namely in the way the *vegan* functions become appropriately adapted for execution within the LifeWatchGreece Cluster Infrastructure. We next describe first the general approach followed for each individual *vegan* function, in order to determine the optimization techniques that should be followed and then we explain in detail our generic methodology for optimizing functions.

Optimization methods focus on three major processes: a) Parallelization, b) Data manipulation through primary and secondary storage and c) Load balancing.

We focus on the parallelization of functions at two levels of abstraction: Level 1 (low level) - Primitive operations like outer product, matrix multiplication, etc, available in the core R package, are addressed at low level using basic mathematical operations, due to their frequent usage. Level 2 (high level) - More, general R functions, like those in *vegan* CRAN package are addressed at the higher level of abstraction, namely job segmentation. Both levels can be combined to reach optimal solutions and achieve significant speed-up. Often the output of a certain function can be utilized as the input of another functions. For such sucessive function executions, level 2 parallelization allows for efficent data portability between functions. (eg. *taxa2dist* -> *taxondive*).

Some issues which we needed to address for this work entailed the general nature of R as a programing language. R is a single-threaded language, so we had to find alternative methods to overcome memory barriers and perform big data segmentation, as well as perform task segmentation using the multi-core system available by a cluster computing environment.

The above issues were addressed primarily using MPI. MPI is a powerful, low-level tool that can provide numerous solutions for R parallelization. It provides a framework for managing communications, while the general process for utilizing MPI in SPMD (Single Program Multiple Data) can be summarized with the following steps: i) Initialize communicator(s), ii) Data input to individual processes, iii) Perform computations, iv) Communicate results, v) Shut down the communicator(s).

In order to perform MPI manipulation for RvLab we adopted *pbdMPI* as our primary package and couple it with other parallelization solutions or code optimization. MPI is simplified through *pbdMPI*, whereby a single program is written and later spawned by mpirun. pbdMPI allows for spawning and broadcasting from within R under a simplified API for all functions, permitting very fast communication.

Moreover, we utilized *pbdR* for big memory manipulations and in conjunction with pbdMPI we achieve low-level and custom parallel solutions and also allows us to benefit from Single Program Multiple Data.

**Performing profiling techniques**

In order to profile for bottlenecks (parts of the algorithm where large amounts of runtime are consumed and greater size of memory is allocated) we combine a variety of functions from several profiling packages, like *profr* (Wickham 2014) and *proftools* (Tierney and Jarjour 2016). Functions like *Rprof*() (for memory profiling) trace parts of the code with greater memory allocations while *proftable*() and *lineprof*(), offer profiling of R code on a line-by-line basis.

**Optimization steps and approach**

Before starting our parallelization methodology, we perform some preliminary tasks in order to distribute efficiently our effort. The first task concerns the application of profiling techniques so as to detect chunks of the algorithm according to memory consumption and computational time. Thereafter, we classify these chunks from most to least demanding. The second task requires categorization of these chunks according to their repetitions inside the code. Functions which contain repeated parts, like often usage of primitive functions, must be treated differently than functions with non-repeated occurances. A choice of low (level 1) or high (level 2) level optimization is then taken depending on whether the function contains repeated occurrences of primitive function operations or non-repeated occurrences, where parallelization of the functions is performed.

The next checkpoint in the workflow depends on whether the size of the data generated by the function operations exceeds the available system RAM capacity. If the data surpasses the available RAM, we use *RPostgreSQL* and *dplyr* packages. These packages allow us to interact with external database, like *PostgreSQL*, in order to overcome the memory barrier. Thereafter, we combine the operation with the *pbdMPI* package to parallelize our function. After the expiration of the above technique we generate and retrieve the desired optimized results. In cases where available RAM is sufficient, we limit our process to *pbdMPI* package usage in order to decrease computational time and to optimize our results.

**Figure 2.**
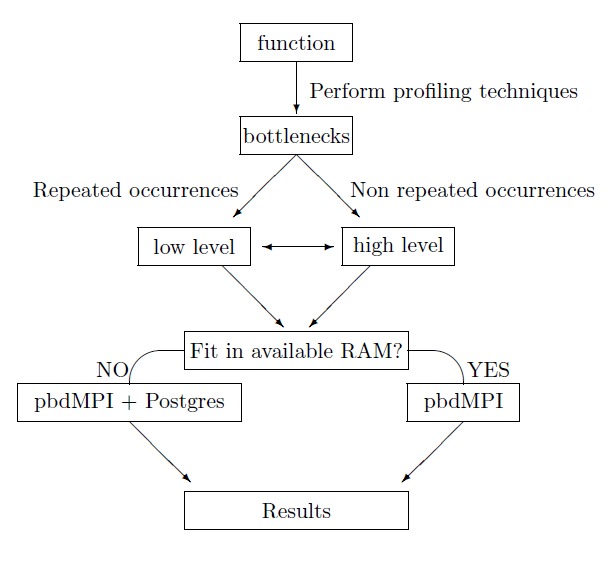
The overall pipeline for the optimization process.

Finally, we reconstruct our results in the appropriate format and we store them form further use or we printed on the screen. The overall pipeline for the optimization process can be seen in Fig. 2.

**Figure 3.**
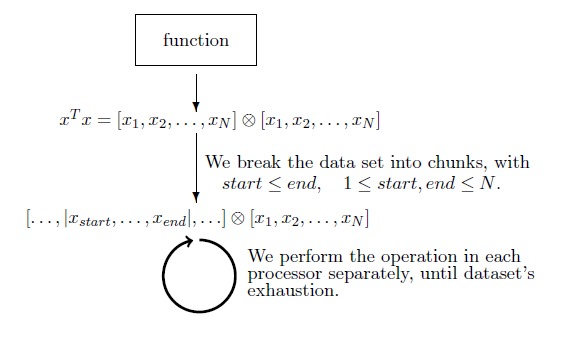
Example of Low level optimization (level 1) applied to multiple and recurrent primitive functions.

**Example 1 Low level optimization (level 1)**. One characteristic example is the parallelization of functions, such as the outer product (Suppl. material 1). The methodology followed is shown Fig. 3, aiming at allogating smaller portions of the work on different processors (Suppl. material 2). A similar methodology is applied on other recurrent primitive functions. The true optimization power of this example code becomes evident upon a high number of repeated executions of this code in our algorithms.

**Figure 4.**
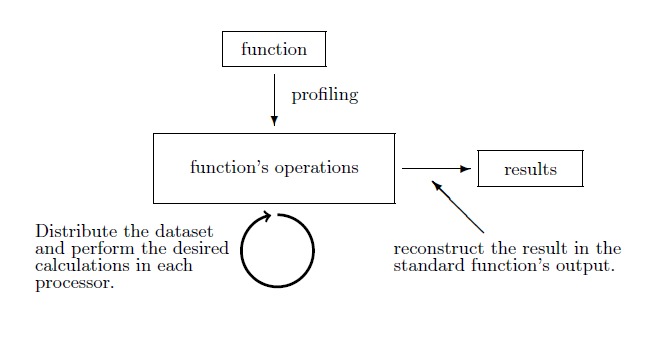
Example of High level optimization (level 2) applied in general functions.

**Example 2 High level optimization (level 2).** In the example shown in Fig. 4 we demonstrate the methodology we apply in a general function. As we seek to enhance its performance we distribute our dataset and we run our code simultaneously for this distributed dataset.

**Generic Methodology**

Our methodology for optimization aims to combine the solutions on the parallelization level with those on the database storage aspect in a harmonious manner, and not just to integrate them monolithically. More importantly, our methodology needs to be flexible enough to be adapted to the different requirements of each function. For example, it is expected that certain functions perform computationally intensive tasks on small datasets, while others iterate simple operations on big datasets or produce bi-products during their computation that are difficult to maintain in the main memory.

**Figure 5.**
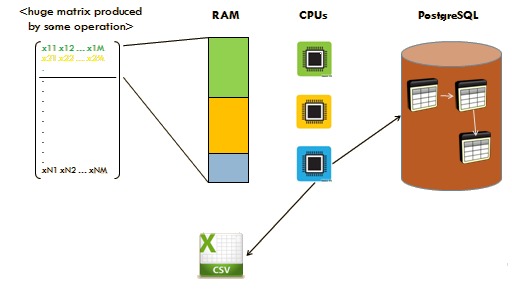
Methodology adopted for operations with memory leakage.

Fig. 5​ presents schematically the general rationale of our methodology. Whenever computations are too demanding and/or too big to fit in main memory (RAM), they are broken down into chunks that can fit in memory. At the time of execution of a job by the cluster, each available processor is assigned to perform a part of the necessary computations. The outcome is then stored temporally in corresponding tables in the PostgreSQL database. The next chunk is brought into memory to repeat the process. If the data in the database is the final outcome of the function, as is the case with the *taxa2dist* function, the tables can be reconstructed and stored in a Comma Separated Values (.csv) file format. If, on the other hand, these tables are only some bi-product generated during computation, they are retrieved part by part and aggregated to carry on with the execution.

It is important to note that the type of computations performed in each processor is not necessarily restricted to primitive operations, such as matrix multiplication, outer product etc. These operations have of course been redesigned to take advantage of the decomposition of data to the processors available. Still, a big asset of the RvLab offered functionalities is that they provide optimizations at higher levels of abstraction, such as in combining sequences of commonly executed *vegan* functions into a single one.

For instance, a commonly executed workflow performed by biologists is to provide the output of the *taxa2dist* function, which is usually a big square matrix, as input to the *taxondive* function, which generates results of a small size. Both of these functions perform executions of similar operations on the same data multiple times; our enhanced function combines these two functions with a more efficient parallel algorithm that not only achieves significantly quicker execution times, as evidenced in a following section, but also overcomes the memory barriers that exist when the initial datasets are beyond a certain limit. In fact, since the output of *taxa2dist* does not need to be stored, our function can be used with input data of any size.

**Supported optimized RvLab functions**

A non-exhaustive list of supported RvLab functions is presented below:

· *taxa2dist* parallel, *taxa2dist* (local storage), *taxa2dist* > *taxondive* - The *taxa2dist* function returns a distance matrix from a classification aggregation file which acts as input for *taxondive*. The combination of these functions computes indices of taxonomic diversity and distinctness, which are averaged taxonomic distances among species or individuals in the community (Clarke and Warwick 1998, Clarke and Warwick 1999).

· *anosim* - Analysis of similarities (ANOSIM) provides a way to test statistically whether there is a significant difference between two or more groups of sampling units. It is often used as a hypothesis test after multidimensional scaling analysis.

· *adonis* - Analysis of variance using distance matrices in order to partition them among sources of variation and fitting linear models (e.g. factors, polynomial regression) to distance matrices. It uses a permutation test with pseudo-F ratios and it is the equivalent of PERMANOVA analysis (Anderson 2001).

· *mantel* - Function *mantel* calculates the Mantel statistic as a matrix correlation between two dissimilarity matrices, and function *mantel.partial* computes the partial Mantel statistic as the partial matrix correlation between three dissimilarity matrices. The significance of the statistic is evaluated by permuting rows and columns of the first dissimilarity matrix.

· *simper* - Returns a list of variables (e.g. species) that contribute to the average similarity within and average dissimilarity between groups of samples, using Bray-Curtis index or Euclidean distances.

· *bioenv* - Returns the best subset of environmental variables, so that the Euclidean distances of scaled environmental variables have the maximum (rank) correlation with community dissimilarities.

The rate statistics are computed using the formula in Suppl. material 3.

### Funding

This work was supported by the LifeWatchGreece infrastructure (MIS 384676), funded by the Greek Government under the General Secretariat of Research and Technology (GSRT), ESFRI Projects, National Strategic Reference Framework (NSRF).

## Web location (URIs)

Homepage: https://rvlab.portal.lifewatchgreece.eu/

## Technical specification

Programming language: R, Javascript, PHP, C#

Operational system: Windows or Linux or Mac

Interface language: HTML, CSS, Javascript

## Usage rights

### Use license

Other

### IP rights notes

MIT license

## Implementation

### Implements specification

**Figure 6.**
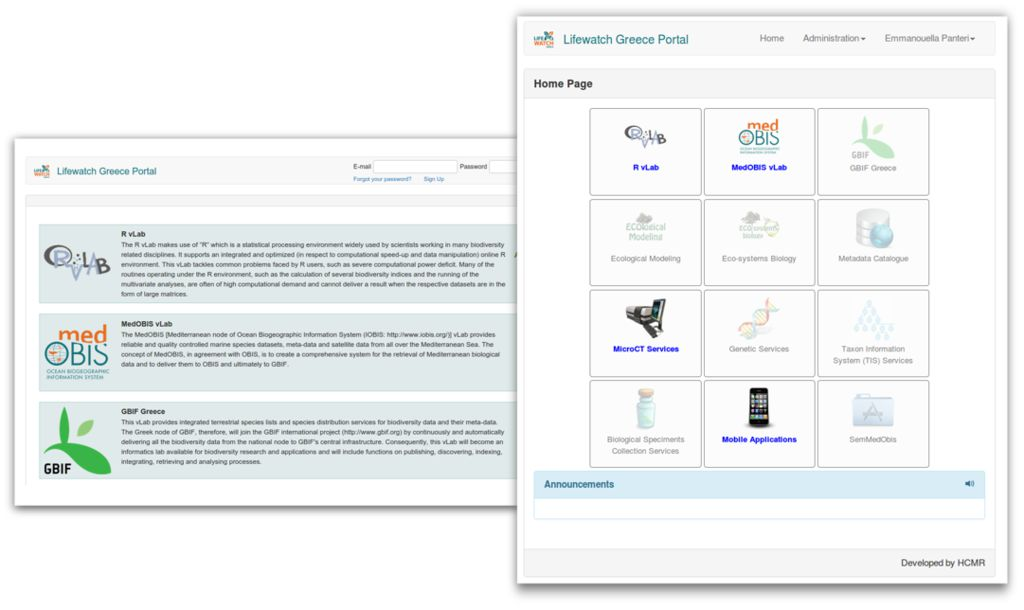
Screenshot of portal's landing (left image) and home page (right image) available via the LifeWatchGreece portal, displaying basic information on all virtual laboratories (including RvLab).

In this section we describe the RvLab web application and how a user can access it through LifeWatchGreece portal. The following screenshots illustrate the web pages a user goes through when using RvLab. After registering and logging in at portal's landing page (Fig. 6 - left image), the user comes to portal's Home Page (Fig. 6- right image) where direct access to RvLab (and other virtual laboratories) is available.

**Figure 7.**
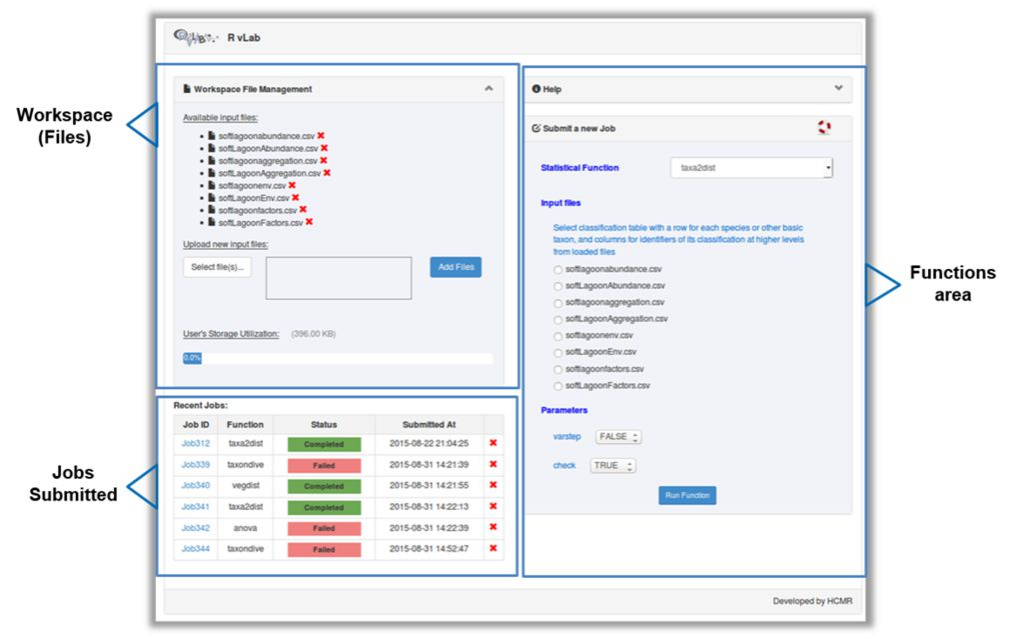
The RvLab main interface.

The main interface of RvLab is comprised by four panels (Fig. 7). The Workspace panel (top left) allows users to upload .csv files that can be used as input and monitor their available storage space. The Functions panel (right) allows users to select a statistical function, configure it and submit a new job to run for execution. The Jobs panel (bottom left) allows the user to keep track of his submitted jobs, monitor their status, view the results or delete the ones that are not needed anymore. The Help panel contains information about RvLab and its usage policies. Example datasets (Fig. 8) can also be found there, if someone wants to try out RvLab without using his own input files.

**Figure 9.**
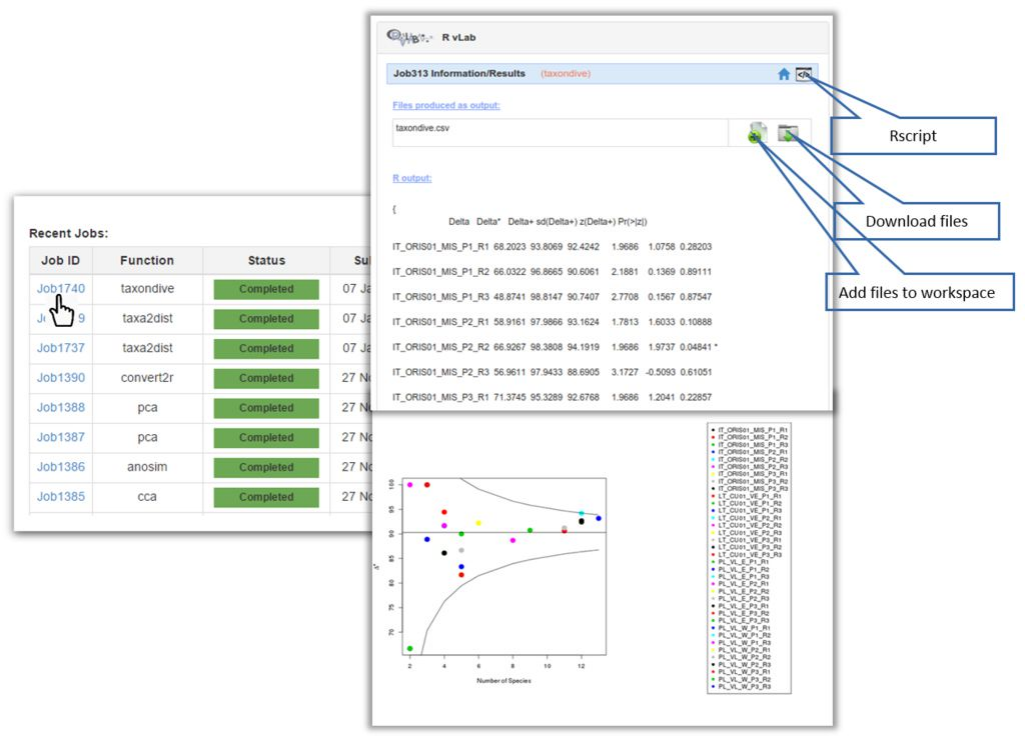
The results page of RvLab.

Once a job is completed, the user can view the results page by clicking on the Job ID link (Fig. 9 - left image). The result page (Fig. 9 - right image) may contain textual information (i.e. "Significance values and taxonomic indices values"), graphical results (i.e. "Taxonomic indices funnel plots") or file results. Result files can be downloaded or added to the workspace for downstream analyses and further utilization in additional RvLab functions.

Screen shots showing examples of the graphical results generated by RvLab can be seen in Fig. 10. The interactive *SUMMARIZEplot* function, utilizing JavaScript Data Drive Documents (D3.js) and HTML (Fig. 10a) allows users to observe distributions of species per station in bar charts, as well as pie charts, generated from the most abundant species found in each station. The results of regression analysis, i.e. the linear model relationship between environmental factors measurements during sampling, such as maximum depth and bathythemetry, are shown in Fig. 10b. Outputs of Analysis of Variance (Anova) (Fig. 10c, left image) and Analysis of similarities (Anosim) (Fig. 10c, right image) can be used to provide statistical significance for the relationship between environmental factors selected by the user. Finally, Principal Component Analysis (PCA) showing an ordination (grouping) of stations in a lower dimensional space, given their species abundance, is presented in Fig. 10d. The user is given flexibility in assigning colour-codes according to selected factors, such as the location of the sampling stations.

RvLab is developed in Hypertext Preprocessor (PHP) and has been integrated in the LifeWatchGreece portal allowing registration to the common user database used for all virtual laboratories available via the portal. This integration utilizes some background Hypertext Transfer Protocol (HTTP) communication between the portal's core and RvLab's web application that involves exchanging information regarding credentials and access control privileges. Moreover, cron jobs have been deployed to ensure that policies are enforced and job status is updated regularly through Asynchronous JavaScript and XML (AJAX) calls. Although job execution takes place on a cluster, RvLab has direct access to all job folders by mounting (through SSH) the rellevant cluster directories to the web server. RvLab uses Portable Batch System (PBS) scripts to schedule each job for execution on the cluster.

**Mobile RvLab application (mobRvLab)**

The RvLab mobile application (mobRvLab) has been developed by utilizing Unity3D Platform and C# scripting language. The application is available for android and ios platforms and functions on a dynamical and autonomous basis. It receives data in json format from the LifeWatchGreece portal in real time by exploiting the appropriate web services that have been developed. Whenever data are required, a secure proper communication channel is established between the device and the portal.

As previously mentioned, the RvLab is available after registration and login to the LifeWatchGreece portal. This is a pre-requisite in order the user to access mobRvLab by utilizing the same account credentials. MobRvLab employs the same functionalities adopted by the RvLab. The mobRvLab main page includes general information about the application and also displays three main tabs: “Functions”, “Files” and “Jobs”. The “Functions” tab shows all available functions, same as in the portal version. The user can select which function to run, including parallel implementations of available functions. In the “Jobs” tab the user can view a jobs log file and keep track of each job status. The jobs are presented and ordered by date. Each job can be selected for viewing results or for deletion. The “Files” tab displays the user workspace and allows file management by uploading or deletion of data files. In principle, the mobRvLab provides a direct link, via mobile access, to the RvLab user account created in the LifeWatchGreece portal; jobs are executed in the LifeWatchGreece cluster and not locally, hence allowing user access to the high performance computational resources of the cluster via mobile appplication. The user can choose to submit jobs and view results from either of the virtual laboratories, benefiting by the usability and flexibility of RvLab in data analysis, as well as by the results acquisition. Fig. 11 displays the data exchange web services and overall functionalities of the mobRvLab.

RvLab mobile application is available for download at https://portal.lifewatchgreece.eu/mobile_apps

### Audience

The desired target audience is the average R user, without requiring expertise and prior training in the field of algorithm optimization and parallelization. However, basic statistical knowledge for using the analytical routines and for the interpretation of results is required.

## Additional information

### Experimental Evaluation

We conducted a series of experiments to evaluate the generic methodology described in previous sections, in order to study (i) the speedup achieved with the new functions when exploiting the resources of a multiprocessor environment and (ii) to identify optimal allocation of resources given the size of the input data. The reported times are the average of 3 runs for each configuration. They were conducted in a controlled environment, where all external access was blocked; despite being an idealized environment, this setting allowed us to reach consistent conclusions about the behaviour of the functions.

**Experimental setup**

Our experiments varied between functions requiring large amounts of memory to handle input data, as well as functions performing computationally intensive tasks. For the former category, datasets of increasing size have been used as input. The *taxa2dist* function, as well as the combined *taxa2dist*+*taxondive*, fall into this category, as they rely on the computation of a distance matrix that can become significantly big depending on the initial dataset. For the latter category, we varied the number of computations, namely the number of permutations that need to be executed before producing the result. For each of these cases, we measured the execution time of the parallel version of our functions when allocating a different number of processors and compared these times to the time needed to run the serial function, i.e., the version provided by the *vegan* package. Note that in certain cases the serial version could not be executed at all, e.g., when the available memory was not enough to handle computations.

All experiments were conducted on a cluster involving 10 Intel Xeon CPU E5-2667 2.9GHz cores with a total of 384Gb RAM. We measured times by allocating 1, 2, 4, 6, 8 or 10 CPUs to the parallel functions, in order to study their behaviour. The results are presented through a series of diagrams which are based on a comparative analysis; absolute timing is mentioned only for verification. Although the current version of the RvLab portal runs over a cluster having a different configuration, the messages conveyed by our experimental analysis are still valid, as we are not interested in the absolute times measured, but rather on the speed up that can be achieved.

**Experiment 1. *taxa2dist***

**Figure 12.**
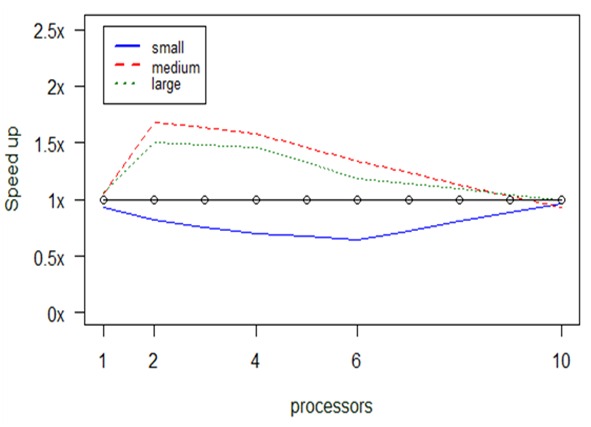
Computational results about optimization in *taxa2dist's* performance, considering the number of processors (x-axis) and the size of the input data.

The diagram in Fig. 12 shows the speedup achieved by running the parallel implementation of the *taxa2dist* function in comparison to the serial one, for datasets having approximately 1,700 lines of species (small), 16,900 lines (medium) and 42,300 lines (large), respectively. Specifically, the black line at 1x sets the boundary in speedup, as it denotes the execution of the serial program itself. Obviously, no matter how many processors we assign to this program, the execution time stays the same since no parallelization of operations is possible within its code. For example, with the small dataset as input, the execution time was 5.1 sec on average with 1 CPU and 5.0 sec with 10 CPUs; for the large dataset, these times where 21.2 min and 21.1 min, respectively. We can safely conclude that the number of processors do not significantly affect the serial program.

Any values below the 1x boundary denote execution times proportially higher to the serial ones, whereas values above the boundary denote how many times faster the execution was found to be. As mentioned above, we measured our parallel program having different allocations of processors, in order to check at which setting the maximum speedup is achieved. In other words, the diagram shows the behaviour (i.e., speedup) of the parallel program in comparison to the serial one (vertical axis) given two parameters, the number of processors assigned to the program (horizontal axis) and the size of the input data (colored lines).

A first observation that can be made is that for small datasets the serial version is somewhat faster than the parallel one. This is displayed by the points lying below the 1x threshold. Indicatively, while the serial version required 5.1 sec on average to run the small dataset as mentioned before, the parallel version with 2 CPUs needed 6.2 sec and with 4 CPUs 7.3 sec. For such small datasets, the differences in absolute times are very small, therefore any delays introduced due to the initialization of the cluster seem to play an important role.

The situation changes as the input dataset becomes bigger, where the parallel version is faster in many cases. For example, with 4 CPUs the parallel version required 2.14 min for the medium dataset and 14.5 min for the large one, whereas the serial version needed 3.4 min and 21.2 min, respectively.

Nevertheless, speedup decreases as we add more CPUs. This observation verifies a conclusion already known in the ICT community: the parallel solution is not panacea and proper allocation of resources needs to be made not only based on their availability, but also on other parameters, such as the size of the input in our case. It seems that the cost of communicating data between processors becomes considerable as we add more processors.

It should be noted here that none of the aforementioned functions was able to operate with datasets of larger size, due to memory overflow. For such datasets, one needs to revert to the PostgreSQL variation that stores data on secondary memory, which inevitably takes much longer times to execute.

**Experiment 2. *taxa2dist*+*taxondive***

Since the output of *taxa2dist* is very often used as input to the *taxondive* function, we implemented the parallel version that combines the two, as described in a previous section. The *taxondive* computes indices of taxonomic diversity and distinctness, which are averaged taxonomic distances among species or individuals in the community (Clarke and Warwick 1998). This function has the added advantage that it manages to overcome memory barriers: due to the algorithmic structure of these functions, one can break the huge bi-products of *taxa2dist* into portions and complete the execution in an incremental manner.

**Figure 13.**
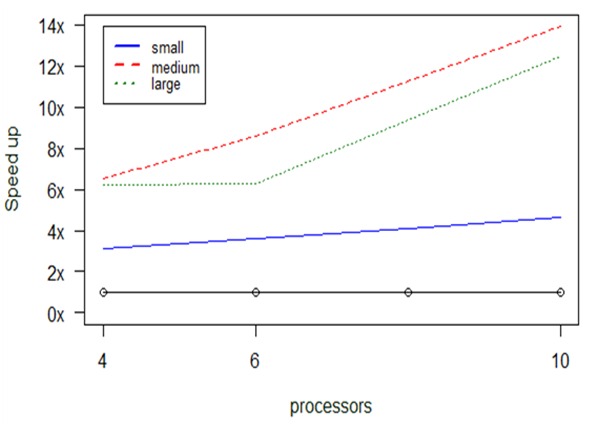
Computational results about optimization in *taxa2dist+taxondive's* performance, considering the number of processors (x-axis) and the size of the input data.

Fig. 13 shows the results obtained, revealing impressive speedups. For instance, while the *vegan* functions require 4.1 hours to execute the large dataset, the parallel version, when exploiting all 10 CPUs of the cluster, manages to reduce this time to 20 min on average. Even for the smallest dataset, where the serial version needs 20.5 sec, the parallel version runs faster, takeing 6.6 sec with 4 CPUs and 4.4 sec with 10 CPUs. One reason for obtaining these results is the more efficient redefinition of the functions made when rewriting the code: we reduced the work needed by avoiding duplicate calculations of operations that exist in the *vegan* (serial) code of both the *taxa2dist* and *taxondive* functions, and we utilized the same structures, wherever possible.

What is even more impressive is that we even managed to run datasets of much larger sizes that cannot be executed otherwise: indicatively, we managed to complete the calculation of the *taxa2dist+taxondive* function for an input dataset having 168,931 lines of species in 6.6 hours when allocating 10CPUs.

**Experiment 3. *anosim***

The next set of experiments concentrated on the *anosim* function. The preliminary profiling tests we performed showed that the main issues one needs to tackle are concentrated on the consumption of time and not on memory overflow. This time consumption is strongly correlated with the number of “permutations” we introduce to the function.

**Figure 14.**
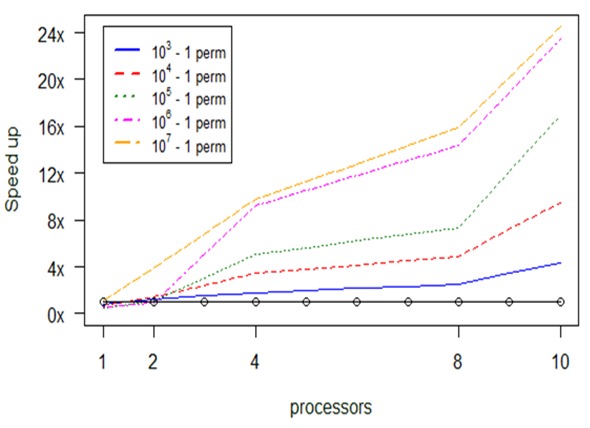
Computational results about optimization in *anosim's* performance, depending on permuations and number of processors (np), for small datasets.

As we see in Fig. 14, as we provide more processors to the parallel version, the execution becomes faster. Another conclusion is that the number of permutations are counter analogous to the algorithm’s running time, but we obtain higher speed up as we increase the number of allocated processors for the increased amount of permutations. This experiment was conducted with a 9 Mb dataset as input .

The above diagram (Fig. 14) suggests that the parallel version with 10 processors completes up to 24 times faster the calculations in comparison to the serial anosim; more specifically, it needs only 1.1 sec as compared to 27 sec. Also, every parallel version is faster than the serial one, apart from the case where the available processors are less than 2. Also, when we have 10^7^ – 1 permutations (orange broken line), the parallel version is faster than the serial.

**Figure 15.**
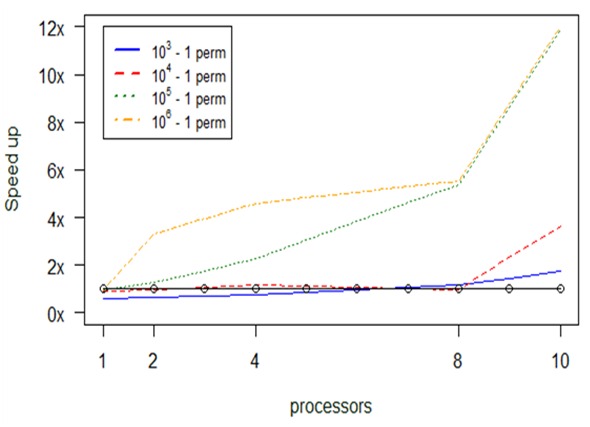
Computational results about optimization in *anosim's* performance, depending on permuations and number of processors (np), for large datasets.

In Fig. 15, for larger datasets, we notice that most parallel versions improve the computational time, except for the case with 10^3^ – 1 permutations and with the number of processors being less than 8. The parallel version with 10^4^ – 1 permutations has neglectable difference with respect to the serial one up until 8 processors. The maximum optimization we achieve is 13 times with 10 processors, from 5.8 min to 28.9 sec. This experiment used a 230 Mb dataset.

**Experiment 4. *adonis***

Our profiling tests with the *adonis* function revealed that no memory issues are likely to be met in this case; instead, the main bottleneck that should concern us is the scaling of computation effort. The dominant factor of time consumption is the number of permutations set as input to the function, therefore we broke the task of computing them into chunks to be assigned to each of the available processors.

**Figure 16.**
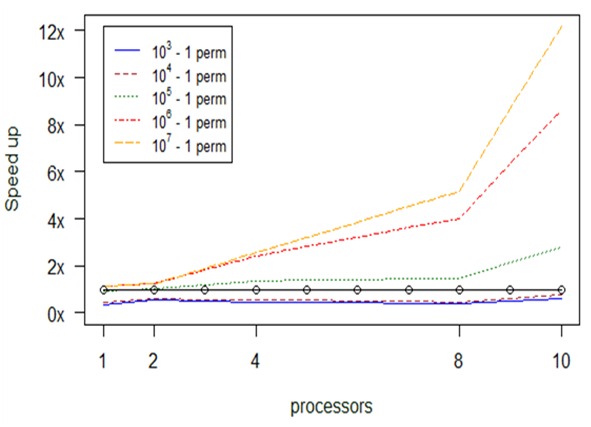
Computational results about optimization in the performance of *adonis*, depending on permuations and number of processors (np).

Fig. 16 shows the speed up gains of the parallel versions. It is evident that the number of processors assigned, directly affects the speed up in each version. Also the number of permutations are counter analogous to the total computation time. As we increase the number of available processors the computational time is reduced up to 12 times compared to the serial version: for example, the serial version requires 8.1 min and the parallel with 10 processors 40 seconds for 10^7^ – 1 number of permutations. Another interesting observation from the diagram is that the execution of parallel computations with a small number of permutations (e.g., less than 10^4^) is not faster than the serial on (black line with circles) despite the resources allocated.

**Experiment 5. *simper***

Similarly to the *anosim* and *adonis* functions, the *simper* function also relies on the number of computations requested and not on the available memory. As before, we break the permutations into chunks which are assigned to the available processors.

**Figure 17.**
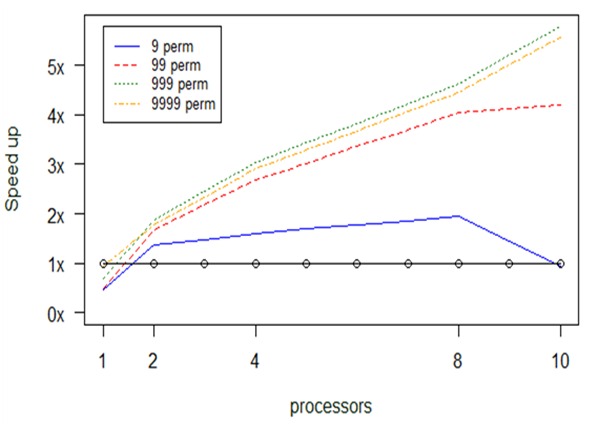
Computational results about optimization in *simper's* performance, depending on permutations and number of processors (np).

Fig. 17 shows the speed up gains implementing the parallel versions. The number of processors is analogous to the speed up in each version. Also the number of permutations are counter analogous to the total computation time, but as we increase the number of available processors the computation time falls up to 5 times compared to the serial version. For instance, the serial version needs 11 sec and the parallel with 10 processors 2 sec, for 10^4^ – 1 number of permutations.

As we already observed in the experiments with the previous functions, we notice again here that when the number of permutations is small parallelization is not always beneficial: in fact, increasing the number of processors may hinder performance, due to the communication costs involved.

**Experiment 6. *mantel***

Similarly to the previous functions, the *mantel* function relies on recurrent computations without producing any memory leakage or overflow. These recurrent computations depend on the number of permutation.

**Figure 18.**
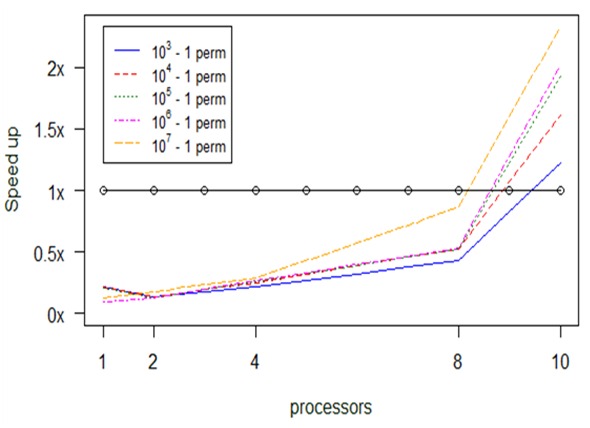
Computational results about optimization in *mantel's* performance, depending on permuations and number of processors (np), for small datasets.

**Figure 19.**
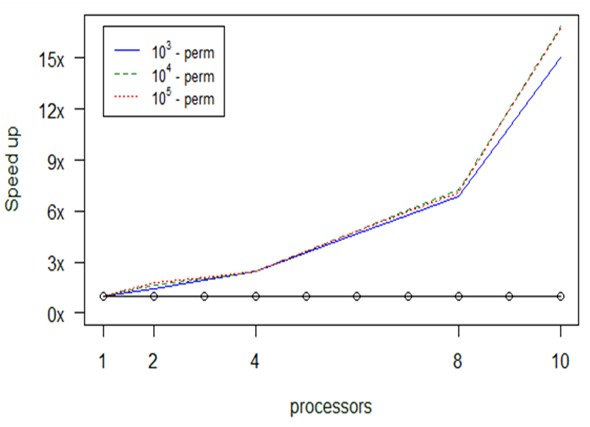
Computational results about optimization in *mantel's* performance, depending on permuations and number of processors (np), for large datasets.

For further investigation we performed two classes of experiments, one for a small input dataset, 9 Mb, and one for a large input dataset, 230 Mb. The diagrams in Figs 18, 19 show that the behaviour of both experiments follow the same principles as the experiments for the previous functions.

In Fig. 18, for the small dataset, as long as we assign less than 8 processors all parallel efforts are less efficient than the serial mantel. Beyond this threshold, we notice a speed up increase and the parallel version outperforms the serial one (black circled line). Finally, using all 10 processors we reach the maximum speed up.

In Fig. 19, for large dataset, all parallel versions improve the computational time. The maximum speed up gain is 15 times, for the parallel version with 10 processors, namely 1.2 sec instead of 20.2 sec for the serial mantel.

**Experiment 7. *bioenv***

*Bioenv* also depends on repeated computations. The main difference with the previous approach is that instead of breaking the permutation’s linked computations into chunks, we break the ncol’s linked computations. This modification came along with a limitation. The limitation suggests that it is not useful to use more processors than the number of ncol.

**Figure 20.**
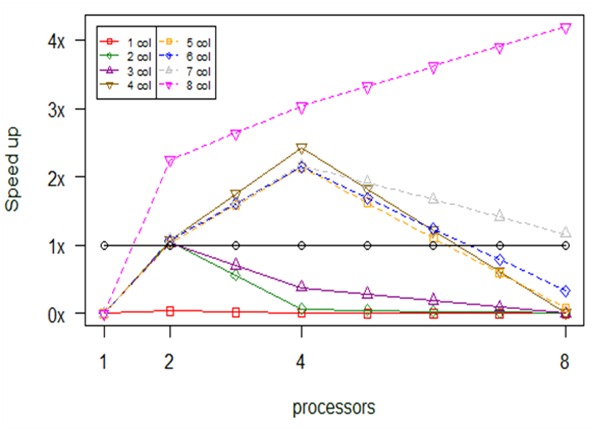
Computational results about optimization in *bioenv's* performance, depending on number of columns and number of processors (np).

In Fig. 20 we report results from an experiment with a dataset with 1 ≤ ncol ≤ 8. As long as we increase both the ncol parameter and the number of processors the speed up increased. The *bioenv* serial version is faster than all parallel versions for ncol = 1. After that, the limitation verified from the results, i.e., the experiment with ncol = 4 reaches 2 times speed up, for 4 processors, with respect to the serial *bioenv*, but if we increase further the number of processors the speed up decreases and converges to 0. A general conclusion drawn from Fig. 20 is that we gain the maximum speed up when the number of available processors becomes equal with the number of ncol. Consequently, the maximum speed up we can gain is about 4 times for the parallel version with 8 processors when ncol = 8, namely we decrease the consumption time from 4.9 sec (serial version) to 1.2 sec (8 processors for ncol = 8).

### Outlook and Future Work

Future implementations of the RvLab will include additional functions that are important for environmental ecology, biodiversity, fisheries and modelling. The RvLab has the possibility to incorporate a variety of functions and R packages, apart from the ones already implemented, should the user contact the development team with a relevant request. Moreover, we are currently also investigating issues of assigning jobs as a function of available resources in order to ensure optimal core distribution and function execution for all jobs submitted to RvLab.

### Conclusions

The RvLab is a very useful and powerful tool, both for users who are already familiar with R (and some of its functions) but also for students and/or scientists who are in favour of open source software and would like to dedicate some time to get familiar with its functions, without having to go through the steep command line R learning curve.

When compared with online virtual environments, such as the "Multivariate AnalysiS Applications for Microbial Ecology (MASAME)" suite, apart from the intrinsic similarities between the two platforms, it is obvious that the RvLab can implement a plethora of functions, some of which are parallelized. Thus, the user can benefit from the availability of newly designed functions if the dataset to be analysed requires their implementation.

The accessibility of RvLab is also one of its major advantages; apart from being part of the LifeWatchGreece Infrastructure, it is also a part of the LifeWatch Marine Virtual Research Environment (VRE). The LifeWatch Marine Virtual Research Environment (VRE) portal is bringing together several marine resources, databases, data systems, web services, tools, etc. into one marine virtual research environment, allowing researchers to retrieve and access a great variety of data resources and tools.

In addition, the RvLab is an interactive virtual laboratory; should the user require other types of functions, these can be added in the "laboratory" and become available online in a short time. Therefore, the more users are logging in the portal and using it for their analyses, the more they can improve the RvLab, given the enormous possibilities of its programming language.

### Appendix

The source code for the functions is available for download at the RvLab.

## Supplementary Material

Supplementary material 1 Matrix 1 Data type: images
File: oo_64064.png Varsos et al

Supplementary material 2 Matrix 2 Data type: images
File: oo_64063.png Varsos et al

Supplementary material 3 Equation 1 Data type: images
File: oo_64065.png Varsos et al

## Figures and Tables

**Figure 8a.**
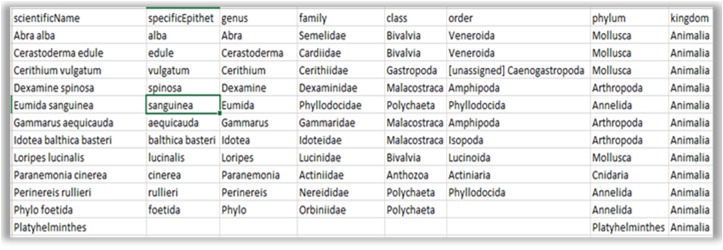
Aggregation file.

**Figure 8b.**
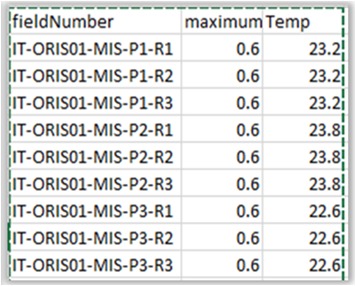
Environmental data file (quantitative).

**Figure 8c.**
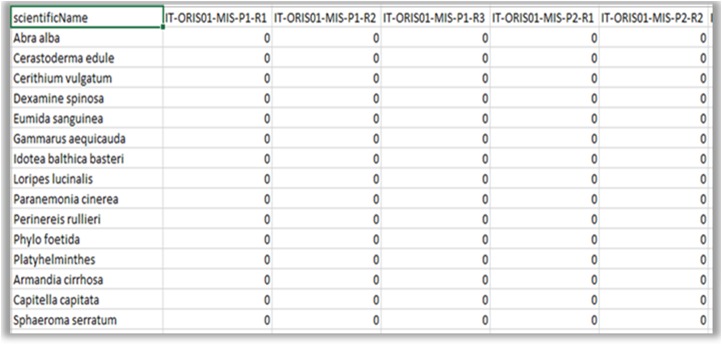
Abundance matrix.

**Figure 8d.**
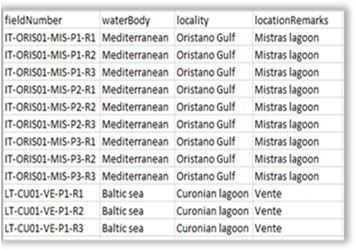
Factor file (qualitative).

**Figure 10a.**
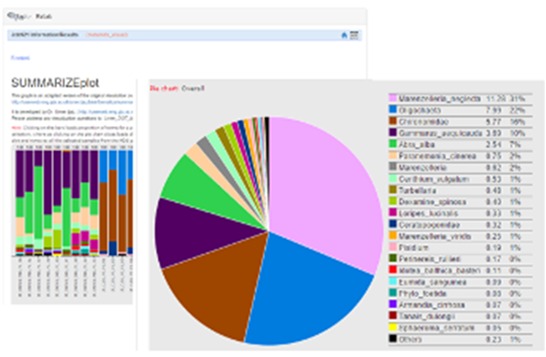


**Figure 10b.**
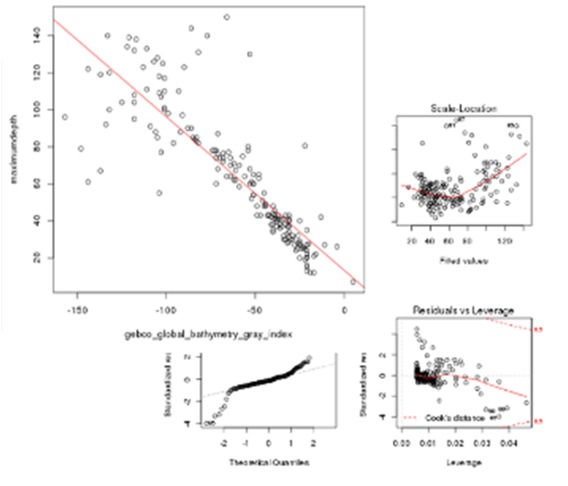


**Figure 10c.**
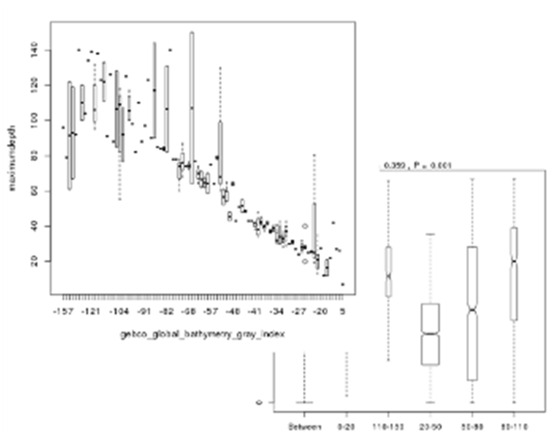


**Figure 10d.**
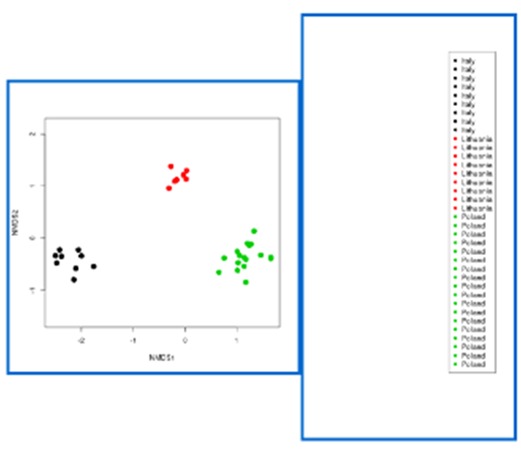


**Figure 11a.**
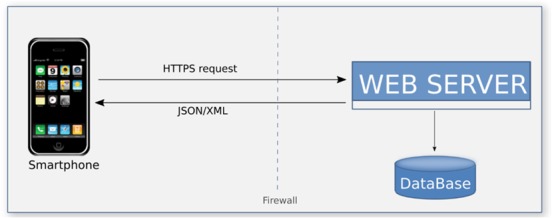
Data exchange web services between portal and mobile RvLab versions.

**Figure 11b.**
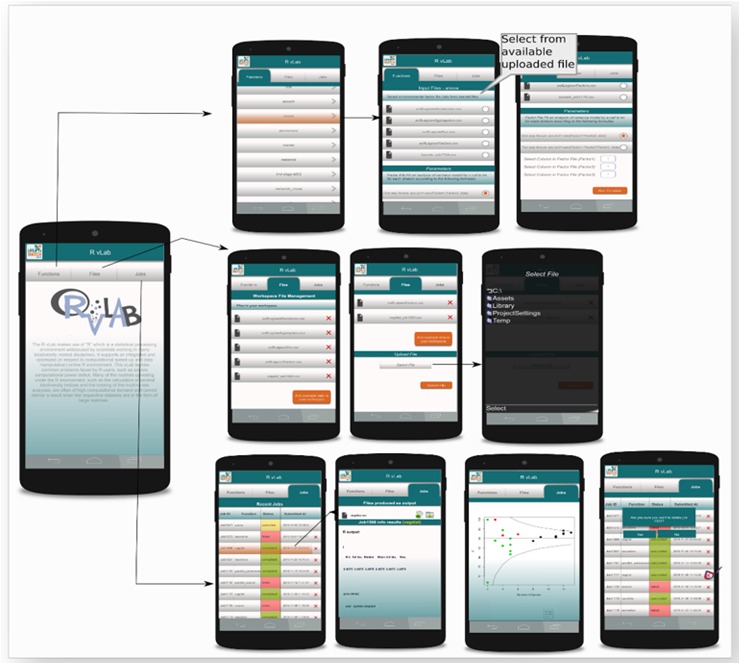
Overall workflow of the mobRvLab interface.
