# Supplementary figures and images for: Optimized R functions for analysis of ecological community data using the R virtual laboratory (RvLab)
Source: Biodivers Data J. 2016 Nov 1;(4):e8357. doi: 10.3897/BDJ.4.e8357 (PMC5136650; doi:10.3897/BDJ.4.e8357)

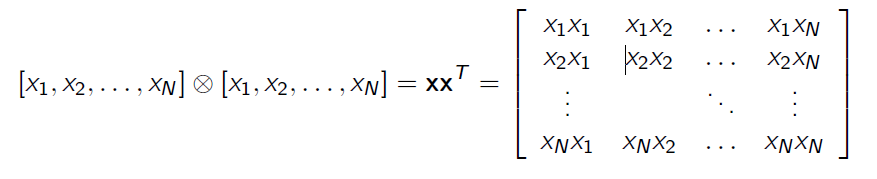

Supplement: Supplementary material 1 — Matrix 1 [file biodiversity_data_journal-4-e8357-s001.png]

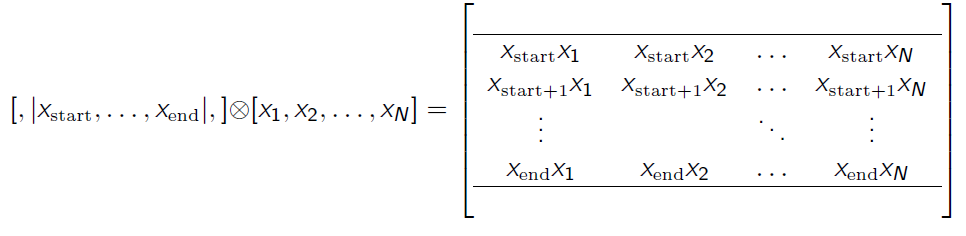

Supplement: Supplementary material 2 — Matrix 2 [file biodiversity_data_journal-4-e8357-s002.png]

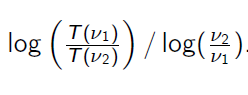

Supplement: Supplementary material 3 — Equation 1 [file biodiversity_data_journal-4-e8357-s003.png]
